# Development of hepatocellular cancer induced by long term low fat-high carbohydrate diet in a NAFLD/NASH mouse model

**DOI:** 10.18632/oncotarget.18585

**Published:** 2017-06-21

**Authors:** Alessandra Tessitore, Valentina Mastroiaco, Antonella Vetuschi, Roberta Sferra, Simona Pompili, Germana Cicciarelli, Remo Barnabei, Daria Capece, Francesca Zazzeroni, Carlo Capalbo, Edoardo Alesse

**Affiliations:** ^1^ Department of Biotechnological and Applied Clinical Sciences, University of L’Aquila, 67100 L’Aquila, Italy; ^2^ S. Salvatore Hospital, Unit of Laboratory Medicine, 67100 L’Aquila, Italy; ^3^ Department of Molecular Medicine, University “La Sapienza”, 00161 Roma, Italy

**Keywords:** NAFLD, NASH, hepatic cancer, LF-HC diet, HF diet

## Abstract

Nonalcoholic fatty liver disease (NAFLD) is a common chronic liver disease. It can progress to nonalcoholic steatohepatitis (NASH) and, in a percentage of cases, to hepatocarcinogenesis. The strong incidence in western countries of obesity and metabolic syndrome, whose NAFLD is the hepatic expression, is thought to be correlated to consumption of diets characterized by processed food and sweet beverages. Previous studies described high-fat diet-induced liver tumors. Conversely, the involvement of low-fat/high-carbohydrate diet in the progression of liver disease or cancer initiation has not been described yet. Here we show for the first time hepatic cancer formation in low-fat/high-carbohydrate diet fed NAFLD/NASH mouse model. Animals were long term high-fat, low-fat/high-carbohydrate or standard diet fed. We observed progressive liver damage in low-fat/high-carbohydrate and high-fat animals after 12 and, more, 18 months. Tumors were detected in 20% and 50% of high-fat diet fed mice after 12 and 18 months and, interestingly, in 30% of low-fat/high-carbohydrate fed animals after 18 months. No tumors were detected in standard diet fed mice. Global increase of hepatic interleukin-1β, interleukin-6, tumor necrosis factor-α and hepatocyte growth factor was detected in low-fat/high-carbohydrate and high-fat with respect to standard diet fed mice as well as in tumor with respect to non-tumor bearing mice. A panel of 15 microRNAs was analyzed: some of them revealed differential expression in low-fat/high-carbohydrate with respect to high-fat diet fed groups and in tumors. Data here shown provide the first evidence of the involvement of low-fat/high-carbohydrate diet in hepatic damage leading to tumorigenesis.

## INTRODUCTION

Nonalcoholic fatty liver disease (NAFLD) is one of the most common chronic liver diseases and the hepatic manifestation of obesity and metabolic syndrome. It can progress to nonalcoholic steatohepatitis (NASH), a more severe form characterized by inflammation, and then to fibrosis and cirrhosis, with consequent severe liver failure and hepatocellular carcinoma (HCC) development in a variable percentage of patients [1, 2]. The strong incidence of obesity, metabolic syndrome and NAFLD is thought to be mainly correlated to consumption of “western diet”, characterized by processed food and sweet beverages [3, 4]. Some mechanisms, such as insulin resistance, peroxidation of lipids, cytokine and adipokine production or oxidative stress, have been described in the NASH pathogenesis [5]. It is known that total high caloric intake is associated to NAFLD/NASH, and several studies have been focused on the role of specific nutrients, such as saturated fat and/or carbohydrate, in the development and transition of the disease [6, 7]. In addition, some studies have described liver tumorigenesis on animal models long term high fat or high fat/fructose diet fed [8–10], but little is known about the specific role of long term high-carbohydrate diet in hepatocarcinogenesis. A work on a NAFLD rat model [11] showed that sucrose, especially when associated to copper deficiency, may promote inflammation, fibrosis and lipogenic pathways. Moreover, some studies, mostly short term, analyzed the effects of fructose on tumor development, showing that it may be a potential risk factor for liver tumorigenesis [12, 13]. Here we used a mouse model predisposed to obesity and NAFLD to analyze long term effects of a low fat-high carbohydrate (sucrose rich) (LF-HC) diet on the progression of liver damage and disease, compared to a high fat (HF) diet. For this purpose, we evaluated the level of steatosis, inflammation and fibrosis as well as the expression of a panel of 15 microRNAs already described in the progression of the disease [10]. Standard diet (SD) fed mice were also analyzed as a control.

## RESULTS

### Body weights

**Figure 1:**
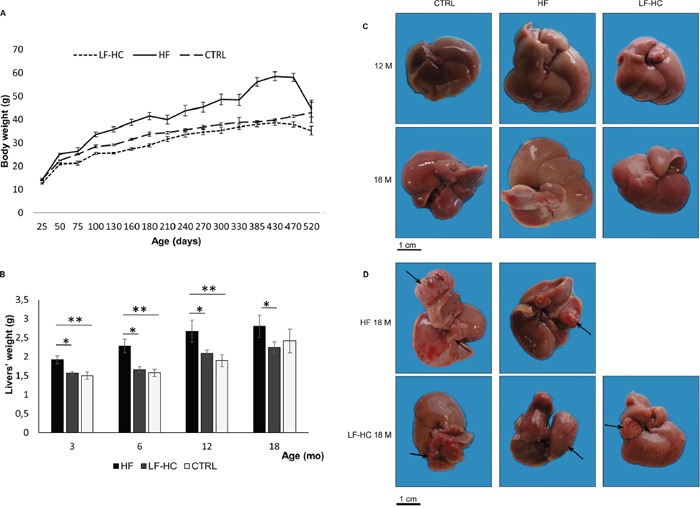
**(A) Body weights.** HF, LF-HC or SD (CTRL) control mice were weighed at the indicated time points (X axis). Values are mean ± SEM. Statistical significance as follows: HF vs. LF-HC P_3M, 6M, 12M_<0.001, P_18M_=0.004; HF vs. SD P_3M_=0.0003, P_6M_=0.0001, P_12M_=0.0006; LF-HC vs. SD P_6M_=0.002, P_18M_=0.013. **(B)** Liver weights, expressed as mean ± SEM. Statistical significance as follows: *, P<0.01; **, P<0.006. **(C)** Livers from 12 and 18 months HF, LF-HC and SD mice. **(D)** Macroscopic nodules on livers from 18M HF (2 out of 5) and LF-HC mice (arrows).

C57BL/6J mice were HF, LF-HC or SD fed for 12 and 18 months. In Table 1 a comparison among diets, principally highlighting the main differences between fat and carbohydrate, is shown. All diets were choline-supplemented. LF-HC and HF diets differ by the sources of fat and carbohydrate (hydrogenated coconut oil and sucrose), whereas show same content of the remaining ingredients (for a complete description of nutrients, please refer to the diet data sheets as reported in Material and Methods). On the other hand, carbohydrate and fat sources in SD were represented by corn/wheat starch and ether extract (see data sheet as indicated in Materials and Methods). Body weights of mice were measured approximately once a month. Significant weight gain was detected in HF with respect to LF-HC fed mice after 3, 6, 12, and 18 months. Significant differences were also detected in the comparison HF vs. SD after 3, 6, and 12 months, and in LF-HC vs. SD after 6 and 18 months (Figure 1A, legend). SD fed mice showed body weight trend included between those obtained from HF and LF-HC diet fed animals. During the last 50 days, weight decrease was observed in HF mice, and, to a lesser extent, in LF-HC mice. Body weight differences were also detectable by comparing 18 months mice with (T) and without (NT) nodules (shown below in “Macroscopic liver features”) (T HF 42.8±2.9, NT HF 46.6±4.4; T LF-HC 32.3±2.5, NT LF-HC 36.2±1.68; weight as mean ± SEM). After 12 and 18 months, subcutaneous, thoracic and visceral fat deposition was observed in all the groups, more markedly in HF mice (data not shown).

**Table 1:** Comparison among diets used for long-term mice feeding, focusing on differences in terms of fat and carbohydrate content (for the comparison of the whole composition, please refer to the data sheets as indicated in Material and Methods)

Ingredient	HF5.56 Kcal/g		LF-HC4.07 Kcal/g		SD3.1 Kcal/g	
	*g%*	*Kcal%*	*g%*	*Kcal%*	*g%*	*Kcal%*
**Protein**	23	16.4	16.8	16.4	18.6	24
**Carbohydrate**	35.5	25.5	74.3	73.1	44.2	58
*Maltodextrin*	*17*	*12.5*	*12.5*	*12.5*		
*Sucrose*	*17.5*	*12.5*	*61.2*	*60.5*		
**Fat**	35.8	58	4.8	10.5	6.2	18
*Soybean oil*	*2.5*	*4*	1.8	*4*		
*Coconut oil, hydrogenated*	*33.3*	*54*	2.9	*6.5*		

### Macroscopic liver features

To define macroscopic appearance, livers from HF, LF-HC and SD fed animals were excised, weighed and analyzed. Livers from HF animals displayed higher volumes than those from LF-HC and SD, and liver significant weight increase was observed in the comparisons HF vs. LF-HC and HF vs. SD. On the contrary, no significant differences were revealed in LF-HC with respect to SD (Figure 1B). Paler color of livers, most probably correlated to steatosis, was evident in HF and, to a lesser extent, in LF-HC with respect to SD diet fed animals (Figure 1C). We previously described voluminous nodular lesions in 2/10 of 12 months HF mice [10]. After 18 months, 50% (5/10) of HF and, very interestingly, 30% (3/10) of LF-HC mice showed macroscopic nodules (Figure 1D). Overall, after 18 months, 7 and 5 macroscopic nodules were detected in HF and LF-HC livers, whose biggest dimensions were included between 0.2 and 1.8 cm in HF, and between 0.2 and 1.5 cm in LF-HC (Table 2). On the contrary, macroscopic nodules were not identifiable in SD fed controls.

**Table 2:** Description of macroscopic nodules in 18 months HF and LF-HC diet fed mice

Mouse ID	Number of macroscopic nodules	Dimensions (cm)
32 HF	2	0,6×0,7×0,3 0,2×0,2×0,2
37 HF	1	1,4×1,8×1,2
38 HF	1	0,3×0,3×0,3
39 HF	1	1,0×1,3×0,8
40 HF	2	0,6×0,6×0,3 0,5×0,5×0,2
35 LF-HC	2	0,8×1,2×0,2 0,2×0,2×0,1
37 LF-HC	1	2,0×1,5×0,7
40 LF-HC	2	0,7×0,5×0,3 0,5×0,3×0,2

### Histological livers’ features

**Figure 2:**
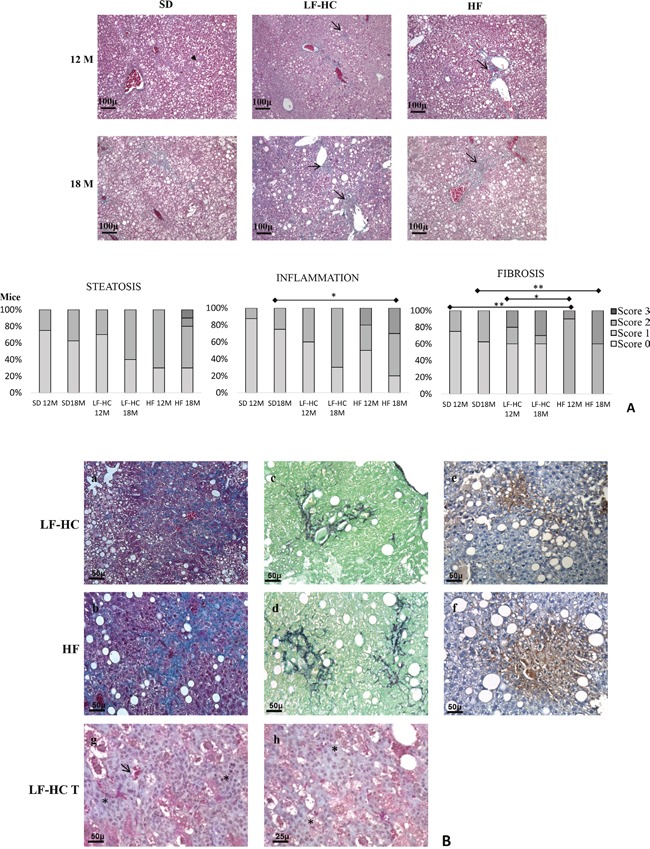
Histological features of hepatic tissues from SD, LF-HC and HF mice **(A)** Masson's trichrome staining (original magnification 10X, scale bar: 100 μm). Steatosis aspects were preeminent in 12 and 18 months HF mice compared to 12 and 18 months LF-HC. In SD mice ballooning degeneration was prevalent (black head arrow). Scattered inflammatory infiltrate (arrows) can be observed in LF-HC and, more severe, in HF mice. Fibrosis, was increasing in LF-HC and HF mice without resembling the typical aspect of cirrhosis. Bottom: graphical representation of the severity of pathological conditions in liver tissues from SD, LF-HC and HF mice. P as follows: * P<0.05, ** P<0.005. **(B)** Masson's trichrome (a,b), Sirius Red-Fast Green staining (c,d) and immunohistochemistry for CD31 (e,f) (original magnification 20X, scale bar 50 μm) of 18 months LF-HC and HF liver tissues. The microphotographs show irregular thin trabeculae (a,b,c,d) and a certain numbers of neovessels with CD31 immunopositivity (e,f). Masson's trichrome staining (g,h) of a macroscopic nodule's section (LF-HC T) from a 18 months LF-HC (original magnification 20X, scale bar 50 μm and 40X scale bar 25 μm) shows an increase of cellular density (asterisk: g,h) with aspects of atypia.

In order to characterize the features and the extent of liver damage, microscopic analysis of hepatic tissues was performed. Progressive diet-dependent liver damage, in some cases significant, was observed at the morphological level in terms of steatosis, inflammation and fibrosis (Figure 2A, 2B). All the liver tissues from SD, LF-HC, HF diet fed animals showed a certain degree of ballooning degeneration, a reversible condition characterized by clear cytoplasm and normal nucleus in central position (Figure 2A). Increasing degree of steatosis severity was revealed by SD, LF-HC and HF diet fed animals after 12 months, and more severe steatosis was progressively detected in the above-mentioned groups after 18 months, as represented in Figure 2A (right, top).

Mild or moderate scattered inflammatory infiltrate, characterized by lymphocytes, plasma cells, macrophages and polymorphonuclear leucocytes (PMN), was observed in liver parenchyma (Supplementary Figure 1). The presence of inflammatory infiltrate was progressively increasing in SD, LF-HC and HF mice after 12 and, more markedly, 18 months (Figure 2A, right, center). In particular, very mild infiltrate was evidenced in SD fed mice.

Finally, we characterized the extent of fibrosis by using both Masson's trichrome and Sirius Red-Fast Green staining. Delicate strands of collagen fibers, close to a variable number of small vessels, were identified. In accordance to that previously described, the severity of fibrosis progressively increased in SD, LF-HC and HF mice after 12 and, markedly, after 18 months (Figure 2A, right, bottom). Very light fibrosis was detected in SD fed mice. Notably, the strands of connective deposition identified did not resemble the typical aspect of cirrhotic liver even in HF mice, and no peri-portally or centrizonally septa were present (Figure 2A, left; Figure 2B, a,b,c,d).

Hepatic tissues from non tumor (NT) or tumor (T) bearing mice were also compared. Despite differences concerning the number of mice belonging to T or NT groups, a more severe trend in terms of steatosis, inflammation and fibrosis seems to be displayed by tumor-bearing mice (Supplementary Figure 2).

Nodules identified at the macroscopic level were excised and microscopically analyzed. Features of dysplastic or early hepatic cancer were evidenced (Figure 2B, g,h). In addition, we observed connective deposition characterized by irregular thin trabeculae that bordered nodules with a variable number of small microscopic vessels expressing CD31 immunopositivity (Figure 2B, e,f). A certain degree of cytological atypia, rare pseudo-glandular structures were also observed (Figure 2B, g,h).

### IL-1β, IL-6, TNF-α, HGF expression

**Figure 3:**
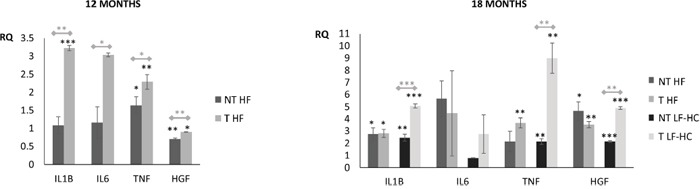
Cytokines and growth factor expression in liver tissues from mice without (NT) and with (T) tumors **(A)** Comparison between pooled RNAs from non-tumor hepatic tissues, obtained from NT and T HF mice. Pooled RNAs from 12 months SD mice livers were used as reference. **(B)** Comparison between pooled RNAs from non-tumor hepatic tissues, obtained from NT and T HF or LF-HC mice. Pooled RNAs from 18 months SD mice livers were used as reference. HPRT was used as endogenous control. RQ: relative quantification. Data expressed as mean ± SE. P as follows: * P<0.05, ** P<0.005, *** P<0.0005. Black stars: significant differences resulting from T/NT LF-HC or T/NT HF vs. SD fed mice. Gray stars: significant differences resulting from the comparison between non tumor NT and tumor T bearing mice.

In order to further characterize that described by morphological analysis, inflammatory cytokines and hepatocyte growth factor expression was assessed by comparing pooled liver RNAs from SD as well as not-bearing (NT) and bearing tumors (T) LF-HC or HF diet fed mice. Results are shown in Figure 3. Significant expression differences were detected by comparing NT or T LF-HC and HF to SD fed mice (black stars). After 12 months, TNF-α increase was detected in both T/NT HF with respect to SD, whereas IL-1β increased only in T HF. Mild, but significant HGF decrease was also identified in both T/NT mice (RQ 0.86 and 0.68, respectively) with respect to SD fed animals. After 18 months, most of the NT and T HF or LF-HC groups displayed significant IL-1β, TNF-α, HGF increase with respect to SD controls. No significant IL-6 differences were detected, although IL-6 increase was evidenced by 12 months T HF, 18 months T LF-HC, T and, to a lesser extent, NT HF compared to SD mice. Significant expression differences were further evidenced by comparing liver tissues from NT with those from T-bearing mice (gray stars): IL-1β, IL-6, TNF-α and HGF increase was detected in 12 months T HF mice; at the same way, IL-1β, TNF-α, HGF increase was shown by 18 months T LF-HC. T LF-HC mice showed in addition IL-6 increase, although this difference was not significant. At the same way, not significant TNF-α increase and IL-6/HGF decrease was shown by 18 months T HF. Overall, these data suggest that liver damage and disease identified in LF-HC mice is similar and only delayed with respect to that detected in HF animals.

### Clinical chemistry assays

In order to further assess liver disease, a panel of plasma biomarkers was analyzed in non-fasting mice. Differences indicating metabolic dysfunctions were detected among the groups (Table 3). After 12 months, significant increase of alanine aminotransferase (ALT) was displayed by HF and, to a lesser extent, by LF-HC mice. Low density lipoprotein (DLDL) increase was shown by HF and, less, by LF-HC mice with respect to SD, whereas ultra high density lipoprotein (UHDL) and triglycerides (TRIG) raised essentially in HF mice. High TRIG levels were also detected in SD. After 18 months, significant DLDL increase was observed in HF and, to a lesser extent, in LF-HC mice with respect to SD. TRIG increase was also detected in HF mice. Aspartate aminotransferase (AST)/ALT ratios were ≥2 in all the experimental groups, with LF-HC values higher than HF (Table 3A). Significant differences were also detected in the comparison (t-test) between HF and LF-HC AST/ALT ratio after 12 and 18 months (P_12M_=0.003; P_18M_= 0.03), ALT and UHDL levels after 12 months (P_ALT_=0.03, P_UHDL_=0.02), TRIG levels after 18 months (P=0.02). In addition, we stratified HF and LF-HC mice in two groups, each developing tumors (T) or not (NT) (Table 3B). Taking into consideration the low number of animals included in each T/NT group (reported in the table), differences were detected. After 12 months, ALT increase was detected in NT HF, whereas higher glucose (GLUC), TRIG, UHDL, cholesterol (CHOL) values were detected in T HF mice. After 18 months, ALT increase was evidenced in T HF and, more, T LF-HC mice. Higher AST levels were displayed by T LF-HC, whereas T HF showed increased DLDL and CHOL levels. TRIG values diminished in both T HF and T LF-HC.

**Table 3:** Chemical chemistry data of plasma from non-fasted mice and AST/ALT ratios, expressed as mean ± SEM

**3A:** Comparisons among 12 and 18 months CTRL, HF and LF-HC mice. Statistical significance was assessed by Kruskall-Wallis test followed by Bonferroni correction (P<0.006). Comparisons were also performed between LF-HC and HF diet fed mice (see text)								
**Marker**	**12 months**				**18 months**			
	**CTRL** **n=8**	**HF** **n=10**	**LF-HC** **n=10**	**P**	**CTRL** **n=8**	**HF** **n=10**	**LF-HC** **n=10**	**P**
ALT U/I	32.7±4.03	89±23	45±6.8	0.002	65.5±19	96±28	101±30	0.626
AST U/I	123.7±24	156.8±24	203±32	0.109	219±27	189±37	318±59	0.147
AST/ALT	2.1±0.7	2.3±0.3	5.1±0.8	0.016	4.2±0.8	2.6±0.3	3.9±0.5	0.078
DLDL mg/dl	9.1±0.6	15.1±1.7	12.6±1	0.012	7.2±0.7	18.7±1.5	15.6±1.4	0.0001
GLUC mg/dl	335.2±30	388±33	398±30	0.389	416±12	356±54	442±29	0.223
TRIG mg/dl	110±6.1	100.7±7.6	82±6.2	0.028	87.7±10.9	108±7.8	71±7.8	0.012
UHDL mg/dl	77.1±2.3	101±7.5	80±2.5	0.025	77.5±1.9	76±10	71.5±5	0.600
CHOL mg/dl	149.6±4.6	208±18.6	173±10	0.144	157±5.7	173±10	166±14	0.992

**3B:** Comparisons among 12 and 18 months HF, LF-HC mice without (NT) and with (T) tumors, expressed as mean ± SEM. ALT alanine aminotransferase, AST aspartate aminotransferase, DLDL direct low density lipoprotein, GLUC glucose, TRIG triglycerides, UHDL ultra high density lipoprotein, CHOL cholesterol						
**Marker**	**12 months**		**18 months**			
	**NT HF** ***n=8***	**T HF** ***n=2***	**NT HF** ***n=5***	**T HF** ***n=5***	**NT LF-HC** ***n=7***	**T LF-HC** ***n=3***
ALT U/I	87.2±28.4	65±6	87.6±31	106±50.6	65.2±15.2	185±83
AST U/I	150±30.3	145.5±1.5	189.2±50	190±60.4	258±46.7	458±145
AST/ALT	2.7±0.8	2.2±0.18	2.6±0.4	2.5±0.5	4.2±0.6	2.8±1.1
DLDL mg/dl	14.2±5.2	14.5±5.5	15.8±1	21.6±2.2	15.5±1.5	15.6±3.7
GLUC mg/dl	348±32	500±52	363.8±51	341±67.3	485±24.3	344±35
TRIG mg/dl	92.4±7.3	120±22	118±14	98.2±5.3	78.5±9.3	53.3±7.4
UHDL mg/dl	90±6.5	123±5	73±14.7	71.5±5	72±7.7	68.6±3
CHOL mg/dl	191.5±22.4	228±26	169.8±34	205.6±44	174±18.5	148±10

### MiRNA expression analysis

**Figure 4:**
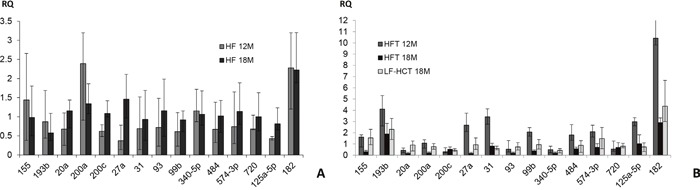
MiRNA expression analysis **(A)** Expression of 15 miRNAs in non tumor hepatic tissues from 12 and 18 months HF vs. LF-HC mice. **(B)** MiRNAs expression in tumors from HF (12 and 18 months) and LF-HC (18 months) mice compared to respective normal HF and LF-HC hepatic tissues. Mamm-U6 was used as endogenous control. All the experiments are mean ± SE of three iterations. RQ: relative quantification.

MicroRNAs play a pivotal role in many fundamental physiological and pathological conditions. We previously identified a panel of 15 microRNAs whose dysregulated expression was described throughout the progression of long term (12 months) diet-induced hepatic damage [10]. Here, we further extended the analysis up to 18 months, by comparing HF to LF-HC non tumor liver tissues as well as tumors to non tumor counterparts (Figure 4). Regarding the comparison between HF and LF-HC non tumor hepatic tissues (Figure 4A), miR-27a expression increase (close to 3 fold), with consequent switch from hypo- to hyper-expression, was detected in 18 months HF livers. Less marked expression increase (included between 1.5 and 1.7 fold) of miR-20a, 200c, 93, 99b, 484, 574-3p and 720 was also detected, with final global iso- expression in HF with respect to LF-HC tissues. MiR-125a-5p expression increase, maintained within the hypo-expression range, was also detected in HF after 18 months. MiR-182 overexpression was revealed by HF livers both after 12 and 18 months. Conversely, miR-155, 193b, 200a level decrease (ranging from 0.5 to 0.7 value) was observed (Figure 4A). Fluctuations were evidenced in tumors with respect to non-tumor liver tissues: after 18 months, miR-27a, 31, 99b, 484, 125a-5p switched from over- to hypo-expression in tumors. MiR-155 and 574-3p displayed similar behavior: they were overexpressed in tumors from 12 months HF and 18 months LF-HC mice, whereas showed downregulation in 18 months HF. Slighter expression changes were detected in miR-20a, 200a and 720. Hypo-expression was maintained by miR-200c, 93 and 340-5p and overexpression by miR-193b and 182 (Figure 4B). Overall, some of the miRNAs here described show more evident modulation among the groups and, for this reason, might be specifically involved in the progression of the disease, depending on the type of diet.

## DISCUSSION

NAFLD is one of the most frequent chronic diseases and is often associated to obesity, a major risk factor for several types of cancer, including that of liver [14]. Its earliest stage is characterized by steatosis which can progress to more severe NASH, with signs of liver cells damage and hepatic inflammation frequently associated to collagen deposition [15]. It is known that a variable percentage of patients affected by NASH can develop hepatic cancer [1]. Carbohydrates are a major stimulus for *de novo lipogenesis*, and some studies have shown that overconsumption of soft drinks and processed foods containing high-fructose corn syrup is associated with liver damage and NAFLD [16, 17]. The main sources of fructose in human diet are high-fructose corn syrup or sucrose from cane or beet sugar. Some studies, focused on explaining the correlation among fructose consumption, insulin resistance and visceral fat in NAFLD, have shown that the sugar induces hepatic and extrahepatic insulin resistance and visceral adiposity [18–21]. Fructose is barely adsorbed by the gastrointestinal tract and cleared by the liver, where it is metabolized with unique mechanism different from that used by hepatocytes for glucose [22, 23]. In the liver, fructose phosphorylation by fructokinase (KHK) generates fructose 1-phosphate which acts as a substrate for aldolase. This produces high levels of ATP and citrate, with consequent fatty acids’ and triglycerides’ biosynthesis. In this context, high fructose diets can cause fat accumulation in the liver and then hepatic damage which can drive to more severe pathological conditions. The previous evidences are supported by a study by Ishimoto et al. [24], where KHK negative mice showed only mild steatosis, without significant inflammation and fibrosis. Cellular and molecular mechanisms linking steatosis, and subsequent pathological conditions, to hepatic cancer have not been clearly defined yet. Some studies identify as major causes of cancerogenesis: a) dysregulated release of adipokines involved in insulin resistance and inflammation [25], b) hyperinsulinemia with hepatocyte proliferative activation and enhanced release of insulin-like growth factor-1 (IGF-1) [26], c) lipid peroxidation leading to oxidative stress and DNA damage [27–29], d) lipotoxicity promoting oncogenesis by PTEN tumor suppressor downregulation [30], e) low grade inflammatory status often associated to obesity, with subsequent increase of specific cytokines (i.e. TNF-α, IL-6) which exert their proliferative and anti-apoptotic effects by activation of the STAT3 oncogenic transcription factor [31]. In this work, we used high fat (HF), low fat-high carbohydrate (LF-HC) or standard (SD) diets for long-term mice feeding to analyze the extent of diet-induced liver damage and disease. Previous studies reported hepatic cancer in long-term HF diet fed mice [9, 10]. Tumor development was in addition observed in a long term study on C57BL/6J mice fed a diet similar to an American fast food diet and enriched with high fructose corn syrup [8]. Here we show for the first time liver tumors in long-term LF-HC diet fed mice, indicating that the hepatocarcinogenesis process may be attributable to high carbohydrate (sucrose) diet. After 18 months, 30% of LF-HC diet fed mice showed nodules with defined neoplastic features. Furthermore, the percentage of HF diet fed animals with hepatic cancer increased from 20% after 12 months to 50% after 18 months, supporting the enhanced detrimental effects due to prolonged HF feeding. Significant weight increase was detected in HF with respect to LF-HC mice, whereas in the last days HF and, to a lesser extent, LF-HC mice showed weight decrease, most probably due to degenerating health conditions as result of the tumors’ development. Accordingly, body weight decrease is further observed in 18 months mice with hepatic nodules with respect to those without. Interestingly, weights of SD fed mice were higher with respect to LF-HC, despite lower SD calorie count, thus suggesting that the adverse health conditions leading to severe liver damage and hepatocarcinogenesis might not merely depend on the total calorie intake or weight gain, but on the specific LF-HC diet components instead. Clinical chemistry data indicate the presence of metabolic dysfunctions, generally less marked in LF-HC and more severe in HF groups, most probably due to the advanced liver disease and possibly depending on the type of diet. Globally, these data confirm metabolic dysfunctions and advanced liver disease in HF and, less, in LF-HC mice. AST/ALT ratio more than 2 was displayed by HF, LF-HC and SD fed animals as well. The highest ratio was shown by LF-HC and this should be specifically investigated in the future. Generally, in humans, AST/ALT in NASH is ≤1, whereas values more than 2 can be attributed to acute alcoholic hepatitis or advanced fibrosis and cirrhosis in advanced chronic liver disease [32, 33]. AST/ALT ratio more than 2 is also reported in fasted 54-56 weeks-old SD/SD+HF diet fed [34] and in non fasted 56-70 days-old SD fed C57BL6/J mice (http://www.criver.com/files/pdfs/rms/c57bl6/rm_rm_d_c57bl6n_mouse.aspx).

After 12 and 18 months, both HF and LF-HC diet fed animals show different degrees of steatosis, inflammation and fibrosis. The severity of these condition is, however, less pronounced in the latter group, indicating that the process of diet-induced liver damage and disease is here just delayed. Expression analysis of inflammatory cytokines and growth factors is in line with that described at the morphological level: in particular, significant differences, more marked after 18 months, of IL-1β, TNF-α and HGF were detected in both tumor and non tumor bearing HF and LF-HC mice with respect to SD fed animals. Significant differences of IL-1β, IL-6, TNF-α and HGF were also detected in the comparison between tumor and non tumor HF and LF-HC bearing mice. Interestingly, pro-inflammatory cytokine enhancement is mostly observable in liver tissues from T-bearing mice, further confirming the role of these molecules in cancer promotion, as already described [28]. The observed HGF increase after 18 months might be correlated to a process of liver regeneration following diet-induced chronic damage [35]. Conversely, just minor percentages of SD fed mice show mild steatosis, fibrosis and very light inflammation, most probably due to old age. Accordingly, deteriorating conditions in terms of liver disease due to aging have been already described in literature [36, 37]. As a consequence, no nodules or abnormal hepatic architecture were evidenced in SD fed animals. We also analyzed the level of expression of several microRNAs, small non-coding molecules able to regulate gene expression at the post-transcriptional level, involved in many fundamental cell processes [38, 39]. Due to their structure and functions, miRNAs are strongly involved in cancer initiation and progression and considered biomarkers as well as potential therapeutic targets in several diseases, including those of liver [40–43]. We already identified and discussed fifteen microRNAs which were modulated (from 3 to 12 months) in hepatic tissues through the transition NAFL-NASH-HCC and in tumors [10]. To complete this scenario, we extended the expression analysis up to 18 months. Most of the miRs globally showed weak expression level differences in liver tissues and/or tumors, indicating that the damage induced in HF and LF-HC diet fed animals is quite similar. Others (e.g. miR-27a, 31, 99b, 484, 125a-5p) showed more pronounced fluctuations: for this reason, they could provide new interesting elements to be further investigated to examine in depth molecular mechanisms involved in liver disease induced by specific diets. Controversial functions of the above-mentioned miRs have been described in the development of hepatic cancer of other types of tumors. MiR-27a seems to act as a tumor promoter [44] or suppressor, with low expression associated to early metastasis in HCC [45]. At the same way, miR-31 overexpression was described in a similar mouse model [9] and, conversely, downregulation in a subset of HCC patients with poorer prognosis [46]. MiR-99b acts in promoting metastasis and in defining poor prognosis in HCC patients [47] or, on the other hand, in suppressing liver metastasis of colorectal cancer by mTOR downregulation [48]. MiR-125a-5p was described as a tumor promoter or a tumor suppressor in cancer [49–53]. MiR-484 is involved in IFN-signaling/microRNA pro-tumorigenic mechanisms and seems to have a crucial role in triggering the liver precancerous process [54, 55]. Finally, miR-182, already discussed and described as early over-expressed in HF with respect to LF-HC mice [10], maintained increased levels in HF non tumor hepatic tissues and in tumors after 12 and 18 months. Due to its stable up-regulation, miR-182 represents an additional interesting molecule to be deeply analyzed in order to shed light on diet-induced hepatic cancer initiation and progression.

In conclusion, results here reported provide, for the first time, evidence about long term LF-HC diet induced hepatic cancer. In the wild-type mouse model we used, tumor development can be promoted by two types of diets which differ about the high content of fat (hydrogenated coconut oil) and carbohydrate (sucrose). This indicates that also long term sucrose intake can induce, just later, pathological conditions similar to those shown by HF fed mice. Contextually, mice fed a standard diet, containing different sources of fat and carbohydrates, show very light inflammatory infiltrate, steatosis and fibrosis, confirming the fact that diet-induced hepatic damage and inflammation is linked to cancer initiation and progression. Inflammatory cytokines and HGF show expression increase in LF-HC and HF mice, especially those with tumors, further sustaining that above-reported. Several dysregulated microRNAs here identified, probably depending on the type of diet and the evolution of the disease, should be deeply analyzed to explain their role in diet-induced hepatocarcinogenesis. This could help to identify molecules to be potentially used in the management of liver illness. Additional experiments, especially focused on analyzing the level of oxidative stress, expression differences of other mediators involved in inflammation, microRNA target genes are needed to further elucidate the molecular mechanisms responsible for liver tumor formation.

## MATERIALS AND METHODS

### Mice treatment

C57BL/6J mice were purchased from Charles Rivers Laboratories (France) and maintained at 21°C on a 12 hours light-dark cycle. Twenty days old male mice, obtained from an established colony, were randomly assigned to 4 groups (10 animals each), and high fat (HF) (5.56 Kcal/g) (D12331, OpenSource, Research Diets) or low fat-high carbohydrate (LF-HC) (4.07 Kcal/g) (D12329, Open Source, Research Diets) diet fed for 12 and 18 months (Table 1). Both diets were choline supplemented. Control mice (2 groups, 8 animals each) were standard diet (SD) (3.1 Kcal/g; fat –ether extract- 18% Kcal, carbohydrate –mainly wheat and corn starch- 58% Kcal) (2018S Harlan Teklad) fed for 12 and 18 months. Mice were weighed at approximately one month intervals and periodically analyzed for signs of disease or morbidity. Animals were sacrificed by CO_2_ asphyxiation, weighed, and head-to-tail measured. Laparotomy was then performed, and livers were visualized and rapidly excised, weighed and photographed. The following parameters were considered: liver appearance, color and weight. Liver tumors were excised, counted and measured with a caliper in their major three dimensions. All experimental procedures involving animals and their care were performed in conformity with national and international laws and policies (European Economic Community Council Directive 86/609, OJ 358, 1 Dec 12, 1987; Italian Legislative Decree 116/92, Gazzetta Ufficiale della Repubblica Italiana n. 40, Feb 18, 1992; National Institutes of Health Guide for the Care and Use of Laboratory Animals, NIH publication no. 85-23, 1985). The project was approved by the Italian Ministry of Health and the internal Committee of the University of L’Aquila. All efforts were made to minimize suffering.

### Histopathology

Specimens obtained from sectioned livers were washed in PBS and immediately immersed in 10% formalin in phosphate buffered saline (PBS) (pH 7.4). Then standard procedures for paraffin embedding were performed. Serial 3 μm sections were stained with Hematoxylin and Eosin (H&E) to assess the liver general architecture and inflammatory infiltrate. Masson's trichrome and Sirius Red-Fast Green staining were also performed in order to detect connective tissue deposition and fibrosis.

We evaluated neovascularization within the liver parenchyma and nodules by immunohistochemical analyses for CD31 (1:50, ab28364, Abcam, Cambridge UK).

Two independent pathologists (AV, RS) performed quantitative analyses.

Steatosis was staged on 0-3 scale as followed: score 0, < 5%; score 1, 5%-33%; score 2, 33%-66%; score 3, >66% (for each specimen: 4 fields, 10X) [56].

Parenchymal inflammation was defined as a focus of two or more inflammatory cells within the lobule. Each focus was counted at 20X magnification (score0=none; 1≤2 foci; 2>2 foci) [57].

Stage of fibrosis was assessed as follow: score 0=none; score 1=perisinusoidal zone or periportal fibrosis; stage 2= perisinusoidal and periportal fibrosis without bridging; score 3: bridging fibrosis; score 4= cirrhosis [57].

The stained sections were then observed by using Olympus BX51 Light Microscope (Olympus, Optical Co., Ltd, Tokyo, Japan), and photographed.

### Chemical chemistry assays

Blood was collected in heparin by cardiac puncture after CO_2_ euthanasia, then plasma was obtained by centrifugation and stored at −80°C. Analyses of biomarkers were performed by using Abbott Diagnostics Architect system and dedicated kits, according to manufacturer's instructions.

### RNA extraction and microRNA expression analysis

RNA extraction and miRNA analysis was performed as previously described [10]. Pooled RNAs from animals belonging to the same LF-HC and HF experimental group were used for miRNA comparisons.

### Expression of cytokines and growth factors

Total non-tumor liver tissue RNA from mice belonging to tumor (T) or non-tumor (NT) bearing mice were pooled together, then 1 μg was retrotranscribed by using the Gene Amp RNA PCR kit (Applied Biosystems), according to the manufacturer's instructions. One microliter of product was used for real-time PCR (2X qPCRBIO SyGreen mix lo-ROX, PCR Biosystems), according to the manufacturer's instructions. HPRT was used as endogenous control. Primers used are reported in Supplementary Table 1. Afterwards, samples were run onto a ViiA 7 instrument (Applied Biosystems). Comparative expression analysis was performed by ViiA7 and Expression Suite softwares (Thermo Fisher).

### Statistical analysis

Clinical chemistry data were analyzed by GraphPad Prism 7 software. Statistical significance was assessed by t-test or Kruskall-Wallis test. RT-qPCR data were analyzed by Expression Suite software (Thermo Fisher) and GraphPad Prism 7.

## SUPPLEMENTARY MATERIALS FIGURES AND TABLES



## Abbreviations

NAFLD: non alcoholic fatty liver disease
NASH: non alcoholic steato-hepatitis
HF: high fat
LF-HC: low fat high carbohydrate
SD: standard diet
CTRL: control
TNF-α: tumor necrosis factor α
IL-6: interleukin-6
IL-1: interleukin 1
HGF: hepatocyte growth factor
STAT3: signal transducer and activator of transcription 3
miRNA: microRNA.
