# Supplementary material for: Development of hepatocellular cancer induced by long term low fat-high carbohydrate diet in a NAFLD/NASH mouse model
Source: Oncotarget. 2017 Jun 21;8(32):53482–94. doi: 10.18632/oncotarget.18585 (PMC5581124; doi:10.18632/oncotarget.18585)
Supplement: Supplementary file 1 [file oncotarget-08-53482-s001.pdf]

Development of hepatocellular cancer induced by long term low fat-high carbohydrate diet in a NAFLD/NASH mouse model

SUPPLEMENTARY FIGURES AND TABLE

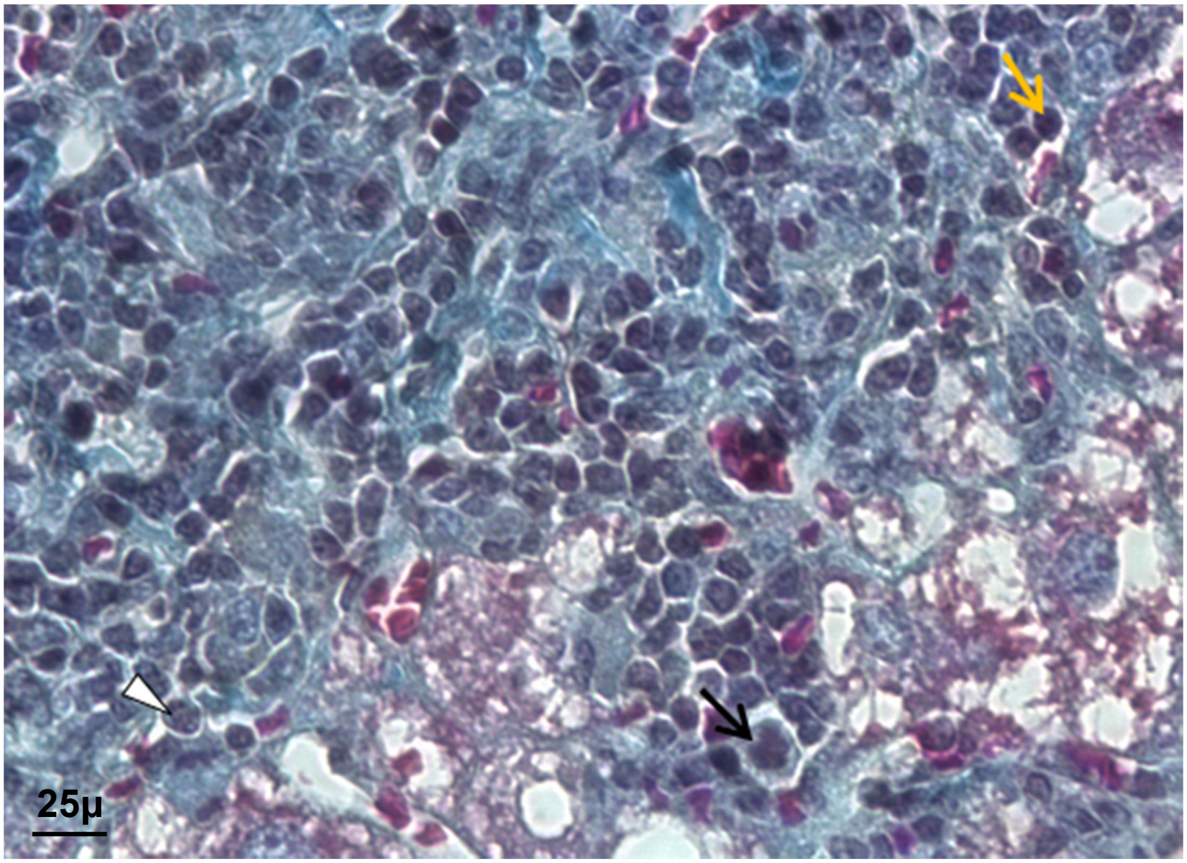

Supplementary Figure 1: Morphological features of inflammatory infiltrate. Masson's Trichrome staining (OM 40X). Inflammatory infiltrate is characterized by lymphocytes (yellow arrow), plasma cells (black arrow) and polymorphonuclear leucocytes (head arrow).

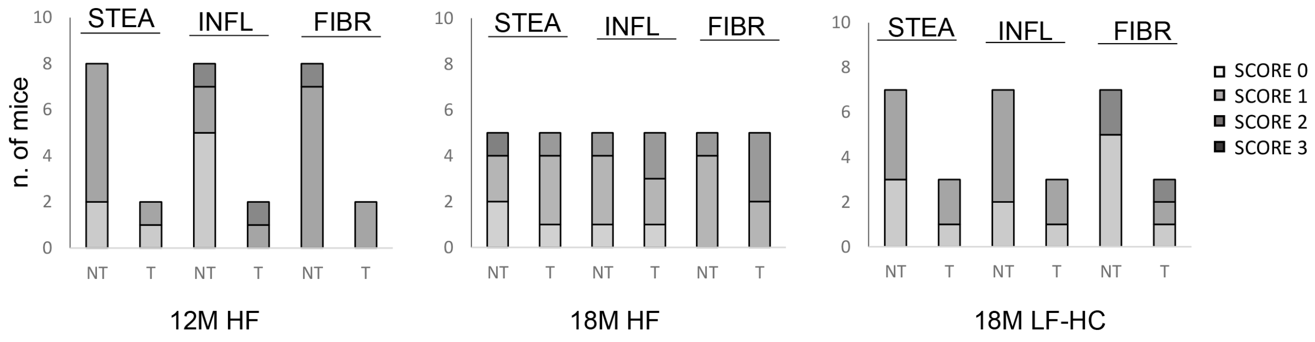

Supplementary Figure 2: Incidence of steatosis, inflammation and fibrosis in non-tumor hepatic tissues from mice without (NT) and with (T) tumors.

Supplementary Table 1: Sequences of primers used for RT-qPCR analyses

IL1β	sense	GAATCTATACCTGTCCTGTG
	α-sense	ACCGTTTTTCCATCTTCT
IL6	sense	CTCTGGGAAATCGTGGAAATG
	α-sense	AAGTGCATCATCGTTGTTCATACA
TNF-α	sense	CCACCACGCTCTTCTGTCTAC
	α-sense	AGGGTCTGGGCCATAGAACT
HGF	sense	TGGGTCTTCCTTGGTAAGAGTAG
	α-sense	CTGCTTCATGTCGCCATCC
HPRT	sense	TTGGATACAGGCCAGACTTTG
	α-sense	TGGCAACATCAACAGGACTC
